# A feasibility study of [18F] FDG PET/CT radiomics in predicting high-risk cytogenetic abnormalities in multiple myeloma

**DOI:** 10.1186/s13550-025-01321-8

**Published:** 2025-10-15

**Authors:** Hong Chen, Jingxin Han, Haozhe Huang, Qi He, Xinqi Ren, Fan Yu, Chunkang Chang, Xuehai Ding, Quanyong Luo

**Affiliations:** 1 Department of Nuclear Medicine, Shanghai Sixth People’s Hospital Affiliated to Shanghai Jiao Tong University School of Medicine, 600 Yishan Road, Shanghai, 200233 China; 2 School of Computer Engineering and Science, Shanghai University, Shanghai, 200444 China; 3 Department of Interventional Radiology, Fudan University Shanghai Cancer Center, Shanghai, China; 4 Department of Hematology, Shanghai Sixth People’s Hospital Affiliated to Shanghai Jiao Tong University School of Medicine, 600 Yishan Road, Shanghai, 200233 China

**Keywords:** Multiple myeloma, Cytogenetic abnormalities, [18F] FDG PET/CT, Radiomics, Machine learning

## Abstract

**Background:**

Multiple myeloma (MM) is a heterogeneous malignancy with prognosis significantly affected by high-risk cytogenetic abnormalities (HRCAs). Traditional detection using fluorescence in situ hybridisation is invasive and limited in capturing disease heterogeneity. We aimed to develop and validate radiomics model based on pretreatment [18F] fluoro-deoxyglucose (FDG) positron emission tomography/computed tomographic (18F-FDG PET/CT) imaging to non-invasively predict HRCAs in newly diagnosed MM patients.

**Results:**

Among the 42 candidate models, the Decision Tree classifier utilizing PET active lesions features demonstrated optimal performance in the validation cohort, exhibiting excellent predictive ability (Area Under the Curve (AUC) = 0.89), significantly outperforming the PET metrics model (AUC = 0.84) and clinical model (AUC = 0.74). SHapley Additive exPlanations analysis identified the PET-derived feature as the most important contributor to the model’s predictive capacity. The model stratified patients into high-risk and low-risk groups, with the high-risk group exhibiting significantly worse PFS and OS (median PFS: high-risk 24.5 months vs. low-risk 29 months; *p* = 0.0360; median OS: high-risk 33.5 months vs. low-risk 50 months; *p* = 0.0023).

**Conclusion:**

As a non-invasive imaging biomarker, PET/CT radiomics holds potential for predicting high-risk cytogenetic status and facilitating patient prognosis stratification Further large-scale, multi-center prospective validations are essential to confirm its utility for personalized therapeutic decision-making in MM.

**Supplementary Information:**

The online version contains supplementary material available at 10.1186/s13550-025-01321-8.

## Background

Multiple myeloma (MM), the second most prevalent hematologic malignancy, is characterized by clonal plasma cell proliferation in the bone marrow, leads to destructive osteolytic lesions, renal dysfunction, anemia, and hypercalcemia [1, 2]. Globally, approximately 588,161 new cases are diagnosed annually and morbidity and younger populations disease burden are also on the rise [3, 4]. Although the emergence of new drugs for multiple myeloma has greatly improved the prognosis, MM has highly heterogeneous clinical outcomes driven by staging, cytogenetic abnormalities, treatments and age [5–8]. Cytogenetic abnormalities —including primary events such as hyperdiploidy and immunoglobulin heavy chain (IgH) translocations, as well as secondary aberrations like del(17p) t (4;14), t (14;16) and gain(1q) —profoundly influence disease course, therapeutic response, and prognosis [7, 9–12]. HRCAs, present in 20–30% of patients, reduce median overall survival (OS) to 2–5 years compared to 7–10 years in standard-risk cohorts, underscoring their critical role in risk stratification and treatment personalization [5, 6, 8, 10, 13]. Despite this clinical urgency, current diagnostic gold standards—fluorescence in situ hybridization (FISH) of CD138-positive cells and genomic sequencing of bone marrow biopsies—remain constrained by invasiveness, sampling bias, and an inability to resolve spatial tumor heterogeneity [13, 14]. These limitations perpetuate unmet needs for noninvasive, systemic approaches to predict cytogenetic risk and guide precision therapies.

As the preferred functional imaging modality, endorsed by the International Myeloma Working Group (IMWG), Fluorodeoxyglucose positron emission tomography/computed tomography (18F-FDG-PET/CT) provides comprehensive multiple meyeloma assessment, demonstrates growing clinical utility in multiple myeloma management, particularly for pretreatment tumor burden assessment through whole-body imaging [14–17]. 18F-FDG-PET/CT enables comprehensive detection of osteolytic lesion distribution, number of focal lesions (FLs), extramedullary disease (EMD), discriminating metabolically active from quiescent myeloma lesions and quantification of metabolic parameters such as maximum standardized uptake value (SUVmax), metabolic tumor volume (MTV) and total lesion glycolysis (TLG)—parameters with established prognostic significance in predicting progression-free survival (PFS) and OS [15, 18–20]. This capability enables critical prognostic stratification for both new diagnosis multiple myeloma (NDMM) and relapsed/refractory disease while facilitating early prediction of therapeutic response. However, conventional metrics like total MTV and TLG, while reflective of global tumor burden, fail to quantify intratumoral heterogeneity—a hallmark of genetic evolution and therapeutic resistance [10, 14].

Radiomics, a high-throughput imaging analysis framework, addresses this gap by extracting advanced textural features (e.g. gray-level co-occurrence matrix entropy, wavelet energy) to decode spatial and metabolic heterogeneity noninvasively and has recently emerged as a tool for early diagnosis, molecular typing, and prognostic evaluation of diseases [21, 22]. Although pretreatment 18F-FDG PET radiomics has revolutionized prognostication and genomic profiling in solid tumors [22, 23], its application in MM remains nascent—currently confined primarily to prognostic prediction and discriminative diagnosis [24–26]. While several investigations have employed spinal MRI radiomics to predict HRCAs in multiple myeloma patients [27–30], the selection of lesions was mostly confined to the spine, and only one study of 31 people used whole-body MRI images [29]. Spatial clonal heterogeneity exists in simultaneously sampled bone marrow sites due to microenvironmental influences [31], which may cause localized imaging cannot fully reflect the lesions information. Whole-body PET/CT imaging overcomes this limitation by comprehensively characterizing spatial heterogeneity across all lesions, EMDs also included. Nevertheless, research efforts utilizing PET/CT-based radiomics for predicting high-risk cytogenetic abnormalities (HRCAs) remain uninvestigated to date [24, 26]. Given the demonstrated superiority of PET/CT in multiple myeloma management and capitalizing on the analytical capabilities of machine learning [32], we propose machine learning models using optimized PET/CT radiomic features, clinical characteristics and PET metrics (MTV and TLG) for predicting HRCAs. By bridging the gap between noninvasive imaging and cytogenetic risk stratification, this approach holds transformative potential to redefine personalized therapeutic strategies in MM.

## Materials and methods

### Patients

This retrospective study was conducted at Shanghai Jiao Tong University Affiliated Sixth People’s Hospital, approved by the Institutional Ethics Committee (Approval No:2024-KY-266(K)) with waived informed consent. Patients diagnosed with active MM per IMWG 2014 criteria between June 2016 and March 2025 were included if they fulfilled the following criteria: (i) pretreatment 18F-FDG-PET/CT (prior to chemotherapy, surgery, radiotherapy, or bisphosphonates); (ii)available FISH results; (iii)age ≥ 18 years; (iv) complete clinical information; (v)hepatic mean standardized uptake value (SUVmean) inside 1.3–3.0 [33]. Exclusion criteria comprised: (i) Poor/inaccessible PET/CT data; (ii) Concurrent malignancy/inflammatory conditions; (iii) Lesion SUVmax < 2.5 or volume < 0.125 cm³.

**Fig. 1:**
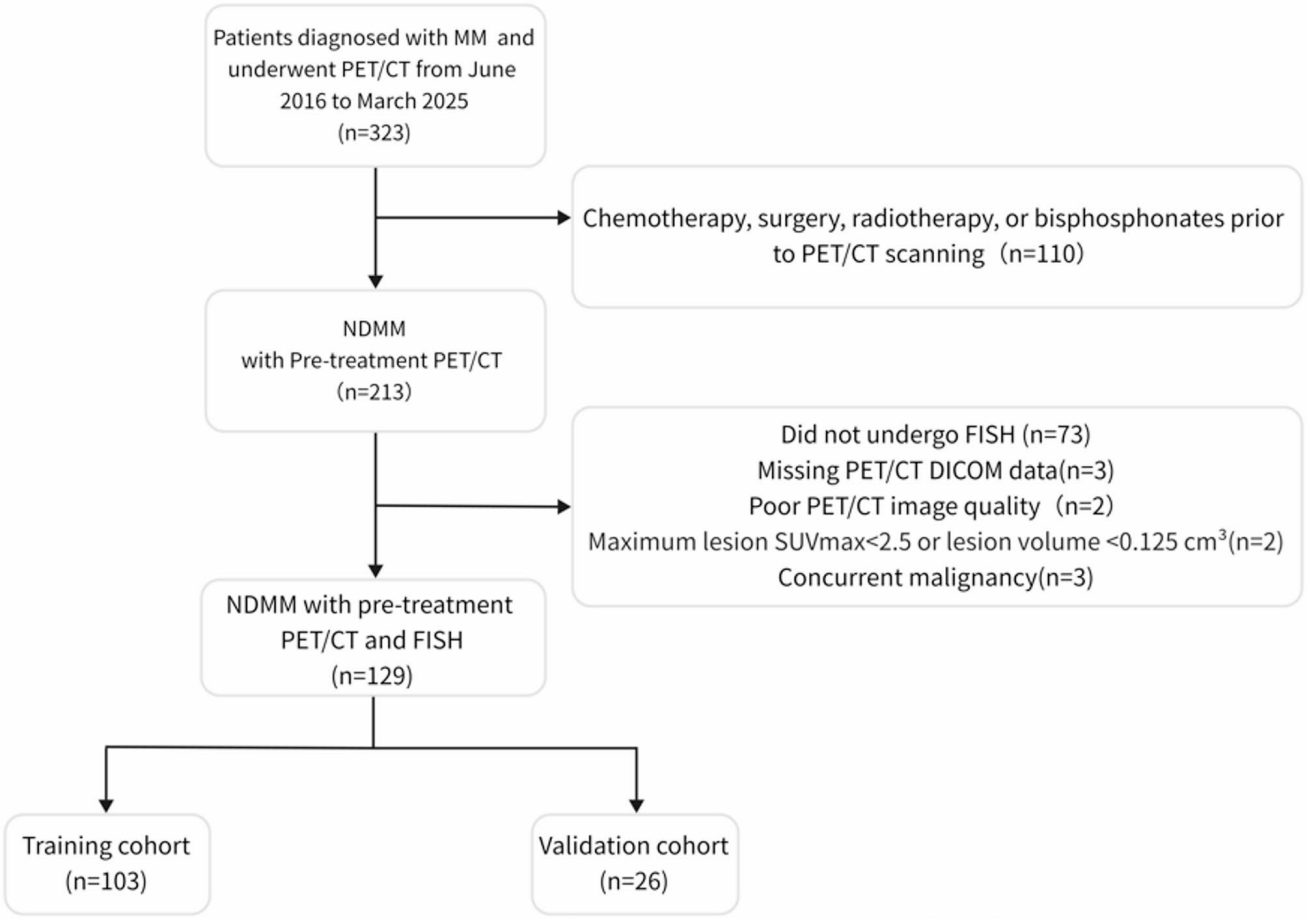
Flowchart of the patient selection process From 323 MM patients with PET/CT scans, 129 newly diagnosed, untreated patients with available FISH and qualified imaging were included. Final cohort was split into training and validation sets.MM, multiple myeloma; PET/CT, fluoro-deoxyglucose (FDG) positron emission tomography/computed tomographic; FISH, fluorescence in situ hybridization; NDMM, new diagnosis multiple myeloma, SUVmax, Maximum Standardized Uptake Value

Ultimately, 129 pathologically confirmed MM cases were enrolled, with a detailed patient selection flow chart provided in Fig. 1. FISH-defined HRCAs included t (4;14), t (14;16),17p deletion (del(17p)), gain of chromosome 1q (gain(1q)) (HRCAs = 67; standard risk cytogenetic (SRC) = 62) [13]. Patients were randomized 8:2 into training (*n* = 103) and validation (*n* = 26) cohorts. Baseline clinical data included demographic, laboratory (hemoglobin (Hb), albumin (ALB), serum lactate dehydrogenase level (LDH), calcium, renal insufficiency (defined as creatinine clearance < 40 mL/min or serum creatinine > 177 µmol/L), anemia (Hb < 100 g/L)), immunoglobulin type, bone marrow plasma cell ratio (BMPC%), International Staging System (ISS) stage, and PET/CT biomarkers (SUVₘₐₓ, MTV, TLG, EMD, FLs). The volumetric metabolic parameters of MTV and TLG were measured using SUV of 2.5 as the threshold from PET-visible lesions (see Suplement1).

### Follow-up and survival assessment

Progression-free survival and overall survival were assessed to evaluate prognostic value of prediction model. PFS was defined per IMWG 2016 criteria as time from diagnosis to progression/relapse/death and OS spanned diagnosis to death [34]. Minimum follow-up durations were stratified by cytogenetic risk: 15 months (high-risk) and 27 months (non-high-risk) based on median survival per FISH analysis. Among 129 patients, 24 with insufficient follow-up or loss to follow-up were excluded (August 2025 is the latest follow-up time), yielding 105 for survival analysis.

### PET/CT data acquisition

PET/CT imaging followed European Association of Nuclear Medicine (EANM) guidelines [33]. After ≥ 6 h fasting and confirming pre-injection glucose ≤ 150 mg/dL, patients received ¹⁸F-FDG (3.7 MBq/kg), followed by one hour uptake phase (50–70 min, mean 60 min). 18F-FDG PET/CT images were performed using a dedicated PET/CT scanner (GE Discovery Max+) including 64 slice CT scanners with a dedicated PET (BGO plus crystal). 18F-FDG images were acquired for 4 min at each bed position from the skull base to the superior mediastinum with patients’ arms along the chest and from the neck to the mid-thigh with patients’ arms above the head. The CT scan was obtained from the orbitomeatal line and progressed to the mid-thigh with the use of a standardized protocol involving 140 kV, 110 mA, 0.8 s/rotation, pitch of 1.75:1, length of scan: 1.0 to 1.6 m, 0.625 spatial resolution, and slice thickness of 3.75 mm. Attenuation correction of PET images was performed using attenuation data from CT and images reconstruction was done using Q Clear reconstruction algorithm with beta = 700. PET intensity was converted to body-weighted standard uptake value (SUV).

### Image preprocessing pipeline

The original PET/CT images are exported by the hospital in DICOM format. Image preprocessing pipeline as flollow: (i) DICOM images were converted to NIfTI format (.nii.gz) using NiBabel (v5.2.1), preserving spatial metadata.(ii)SUV normalization and conversion via pydicom(v2.4.4)​;(iii)Spatial co-registration: PET images were resampled to CT space (voxel spacing/size/direction/origin alignment).(iv) Region of interest (ROIs) Delineation: a)PET Masks: Initial SUV > 2.5 thresholding [33], manually refined in ITK-SNAP software (v3.8.0),excluding physiological/benign uptake. b)CT Masks: Manual osteolytic lesion segmentation (≥ 2 consecutive slices) with extramedullary disease assessment. All regions of interest (ROIs) were manually delineated on each transaxial slice using ITK-SNAP software by two nuclear medicine physicians with 10 (HC) and 5 (FY) years of experience, based exclusively on 18F-FDG PET and CT images, respectively. Any discrepancies between the readers were adjudicated by a senior nuclear medicine physician (QL, with 22 years of experience) through consensus review. All physicians were blinded to the FISH results and clinical outcomes.

### Radiomics analysis and model construction

**Fig. 2:**
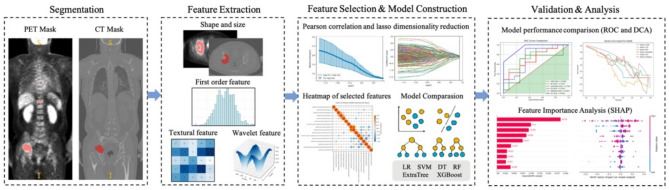
Flowchart of the MM radiomics approach for predicting cytogenetic status (I) Image data region calibration. (II) Extraction of image histology features, including shape and size, first-order statistical, texture, and wavelet features. (III) Feature selection and model construction; (IV) Model evaluation and analysis. MM, multiple myeloma.LR, logistic regression, SVM, support vector machine, DT, decision trees, RF, random forests, ExtraTree, and XGBoost, receiver operating characteristic (ROC) curve, DCA, decision curve analysis

The radiomics analysis was executed through a series of steps: image segmentation, feature extraction, feature selection, model construction, and performance evaluation (Fig. 2). As shown in Fig. 2, the radiomics modeling method of this study is divided into the following key steps. (1) Image data region calibration: Lesion masks (manual/semi-automated) were co-registered across PET/CTmodalities to ensure spatially congruent feature extraction (see Supplement1 PART I). (2) Feature extraction: Multi-dimensional radiomic features—including shape, first-order statistics, texture, and wavelet descriptors—were extracted using PyRadiomics (v3.1.0) [35]. To address PET-CT spatial discordance, five distinct radiomic models were developed by employing extracted conventionally radiomic features from PET and CT: (i) CT model: CT radiomic features derived from CT visible lesions region (ii) CTp model: CT radiomic features derived from co-registered PET visible lesions region, and (iii) PET model: PET radiomic features derived from PET visible lesions region, and (iv) PET&CT model : constructed by utilizing the radiomic features derived from PET visible lesions region and CT visible lesions region of both imaging modalities, separately and (v) PET&CTp model: established by integrating radiomic features from PET visible lesions region of both imaging modalities (3) Feature selection and model construction :1)Radiomics features: (i)eliminating highly correlated features (Pearson’s *r* > 0.9); (ii)the least absolute shrinkage and selection operator (LASSO) regression, coupled with 5-fold cross-validation, was utilized to identify informative features with non-zero coefficients and calculate the corresponding feature weights (λ optimized by minimal mean square error); 2) Clinical features: clinical predictor evaluation through univariate/multivariate logistic regression (reporting odds ratios with 95% CIs);3)PET metrics: using MTV and TLG to constructed MTV&TLG model. Then, six machine learning classifiers—support vector machine (SVM), decision tree (DT), random forest (RF), extremely randomized trees (Extra Trees), XGBoost, and logistic regression (LR)—employed the features filtrated by LASSO feature screening. In total, 42 machine learning models were formulated by integrating the five distinct radiomic model, clinical features and PET metrics with the six machine learning classifiers for predicting HRCAs.

### Model validation and analysis

To ensure the stability of the prediction models, we randomly divided patients into the training cohort and test set with 8:2 ratio. Discriminatory performance was assessed using the area under the receiver operating characteristic curve (AUC-ROC), F1-score, accuracy, sensitivity, specificity, positive predictive value (PPV), and negative predictive value (NPV). Comparative model analysis employed the DeLong test, while clinical utility was evaluated via decision-curve analysis (DCA). Feature interpretability utilised SHapley Additive exPlanations (SHAP) [36], and prognostic stratification was conducted through Kaplan-Meier analysis with log-rank testing.

### Statistical analysis

Statistical analyses were performed using Python (version 3.7.0). Continuous variables were presented as mean ± standard deviation (SD) and compared using the Man-Whitney U test. Categorical variables were expressed as counts with percentages and compared using the Chi-square or Fisher test. Variables with a *P*-value < 0.2 in univariate regression analysis were included in multivariable analysis. The hazard ratio (HR) and associated 95% CI together with the *p* value of the log-rank test were also reported. All statistical tests were two-sided with a significance level set at *P* < 0.05.

## Results

### Patient characteristics

A total of 129 patients(64 male)were included in the study, with age of 64 ± 9.73 years. Among these, 67 patients (51.93%) exhibited high-risk chromosomal abnormalities (HRCAs), while 62 patients (48.07%) had standard-risk chromosomal abnormalities (SRC). In the high-risk group, chromosomal abnormalities included gain of 1q21 (*n* = 50), t (4;14) translocation (*n* = 14), t (14;16) translocation (*n* = 3), and del(17p) (*n* = 9). Of the patients with high-risk abnormalities, 48 had a single abnormality, 11 had two abnormalities, and 3 had three or more chromosomal abnormalities. The training dataset consisted of 103 patients (53 HRCAs and 50 SRC) selected via random division, while 26 patients (14 HRCAs and 12 SRC) contributed the independent test dataset. No statistically significant difference in the distribution of cytogenetic risk status was observed between the training and validation cohorts (*p* = 0.99). Table 1 outlines the clinical and imaging characteristics of patients across both cohorts. Across the training and validation datasets, there were no significant intergroup differences in clinical or imaging features (*p* = 0.1071–0.9508).

**Table 1:** Characteristics of patients between the training and validation cohorts

Characteristics	Training dataset			Test dataset			*P* value(N1vsN2)
	N1 = 103	SRC = 50	HRCAs = 53	N2 = 26	SRC = 12	HRCAs = 14	
Sex							0.861
Male	51	31	20	13	5	8	
Female	52	19	33	13	7	6	
Age	63.8 ± 9.4	63.3 ± 10.3	64.2 ± 8.5	65.4 ± 11.1	64.1 ± 11.7	66.6 ± 10.9	0.2399
Fracture							0.795
No	58	29	29	16	8	8	
Yes	45	21	24	10	4	6	
Osteoporosis							0.727
No	50	29	21	11	5	6	
Yes	53	21	32	15	7	8	
Renal insufficiency							0.565
No	85	43	42	23	12	11	
Yes	18	7	11	3	0	3	
Hypercalcemia							0.314
No	92	43	49	21	10	11	
Yes	11	7	4	5	2	3	
Anemia							0.861
No	52	31	21	12	5	7	
Yes	51	19	32	14	7	7	
LDH abnormality							0.930
No	92	47	45	24	12	12	
Yes	11	3	8	2	0	2	
BMPC ratio (%)							0.930
< 60	92	46	46	24	10	14	
≥ 60	11	4	7	2	2	0	
Albumin							0.591
< 35	43	14	29	13	5	8	
≥ 35	60	36	24	13	7	6	
Serum β₂-microglobulin							0.935
≤ 5500	60	36	24	16	10	6	
> 5500	43	14	29	10	2	8	
Immunoglobulin type							0.798
Non-LC	84	44	40	20	10	10	
LC	19	6	13	6	2	4	
ISS							0.708
1	33	22	11	7	4	3	
2	31	15	16	10	6	4	
3	39	13	26	9	2	7	
EMD							0.787
No	96	46	50	24	11	13	
Yes	7	4	3	2	1	1	
Number of FLs							0.334
≤ 3	12	10	2	5	1	4	
> 3	91	40	51	21	11	10	
Hemoglobin	99.9 ± 26.8	108.6 ± 27.0	91.7 ± 24.2	100.7 ± 25.2	100.8 ± 27.2	100.5 ± 24.4	0.9508
SUVmax	7.4 ± 6.0	7.9 ± 4.9	7.0 ± 6.9	8.8 ± 5.8	7.3 ± 6.1	10.0 ± 5.6	0.1895
MTV	234.0 ± 376.1	183.5 ± 305.8	281.6 ± 429.6	113.4 ± 226.9	199.7 ± 315.6	39.6 ± 44.8	0.1205
TLG	929.2 ± 1624.8	752.7 ± 1391.7	1095.8 ± 1815.2	377.0 ± 805.7	687.0 ± 1122.4	111.2 ± 133.5	0.1071

BMPC, bone marrow plasma cell; EMD, extramedullary disease, FLs, focal lesions, ISS, International Staging System, LDH, lactate dehydrogenase, LC,Light-chain,SUVmax, maximum standardized uptake value ，MTV, total metabolic tumor volume; TLG, total lesion glycolysis，SRC, standard risk cytogenetic, HRCAs, high-risk cytogenetic abnormalities

Continuous variables were presented as mean ± standard deviation (SD) and compared using the Man-Whitney U test. Categorical variables were expressed as counts with percentages and compared using the Chi-square or Fisher test

### Establishment of clinical and PET metrics predictive models

Univariate logistic regression (Table 2) identified multiple factors associated with HRCAs (*p* < 0.2), including serum β₂-microglobulin, hemoglobin, ALB, ISS, sex, anemia, LDH abnormality, Immunoglobulin type, osteoporosis, renal insufficiency, number of FLs (*P* < 0.2). Subsequent multivariate logistic regression analysis revealed serum β₂-microglobulin (OR = 6.312, *p* = 0.029), ALB (OR = 0.205, *p* = 0.010), and immunoglobulin type (OR = 3.342, *p* = 0.034) as independent predictors. These were integrated into clinical models using six machine learning algorithms (Supplement1 Table S8). Among these, the DT model demonstrated optimal predictive performance, achieving AUCs of 0.75 in the training cohort and 0.74 in the validation cohort (see Table 3). The PET metrics model (based on MTV and TLG, Supplement1 Table S9) achieved AUC values of 0.91 and 0.84 in the training and validation cohorts, respectively (as summarized in Table 3).

**Table 2:** Univariate and multivariate analysis of clinical characteristic data

Characteristics	Univariate analysis		Multivariate analysis	
	OR (95%CI)	*P* value	OR (95%CI)	*P* value
Serum β₂-microglobulin	3.546 (1.683–7.471)	< 0.001	6.312 (1.205–33.059)	0.029*
ISS stage	2.028 (1.299–3.166)	0.002	0.557 (0.179–1.738)	0.314
Hemoglobin	0.980 (0.966–0.994)	0.005	0.996 (0.966–1.027)	0.800
Albumin	0.358 (0.174–0.739)	0.005	0.205 (0.061–0.686)	0.010*
Sex	1.929 (0.958–3.884)	0.066	1.792 (0.751–4.277)	0.189
Anemia	1.929 (0.958–3.884)	0.066	0.585 (0.132–2.601)	0.481
LDH abnormality	3.450 (0.903–13.184)	0.070	3.002 (0.635–14.182)	0.165
Immunoglobulin Type	2.295 (0.911–5.783)	0.078	3.342 (1.095–10.197)	0.034*
Osteoporosis	1.799 (0.894–3.619)	0.100	1.342 (0.584–3.083)	0.488
Renal insufficiency	2.075 (0.777–5.544)	0.145	1.840 (0.447–7.582)	0.399
Number of FLs	2.193 (0.758–6.342)	0.147	1.526 (0.414–5.630)	0.526
MTV	1.000 (0.999–1.001)	0.479		
Hypercalcemia	0.687 (0.239–1.972)	0.485		
SUV_max	0.996 (0.940–1.056)	0.899		
TLG	1.000 (1.000–1.000)	0.573		
Fracture	1.200 (0.596–2.415)	0.609		
EMD	0.724 (0.185–2.828)	0.642		
Age	1.014 (0.978–1.051)	0.449		
BMPC ratio (%)	1.089 (0.345–3.438)	0.885		

* Statistically significant difference in multivariate analysis

Univariate and multivariable logistic regression analyses were performed. OR, odds ratio; 95% CI, 95% confidence intervals; BMPC, bone marrow plasma cell; EMD, extramedullary disease, FLs, focal lesions, ISS, International Staging System, LDH, lactate dehydrogenase, MTV, total metabolic tumor volume; TLG, total lesion glycolysis

### Feature importance

For radiomics, a total of 2553 (CT model = 851, CTp model = 851, PET model = 851, PET&CT model = 1702, PET&CTp model = 1702) radiomic features were extracted from PET and/or CT masks respectively (Supplement Table S1), encompassing 14 shape descriptors, 18 first-order statistics, 75 texture features, 600 wavelet-transformed features, and 144 first-order features derived from wavelet decomposition. Pearson correlation coefficients were used to reduce redundancy (threshold > 0.9), and subsequent LASSO regression with five-fold cross-validation identified the most informative features (Supplement Table S1).In accordance with the guideline proposed by Chalkidou et al. —which recommends at least 10–15 patients per radiomic feature to reduce false discoveries [37]—we limited the number of selected features across our models: 8 for the CT model, 8 for the CTp model, 9 for the PET model, 8 for the PET&CT model, and 7 for the PET&CTp model. Further details are provided in Supplement 1, Table S1.

### Performance and comparison of models

**Fig. 3:**
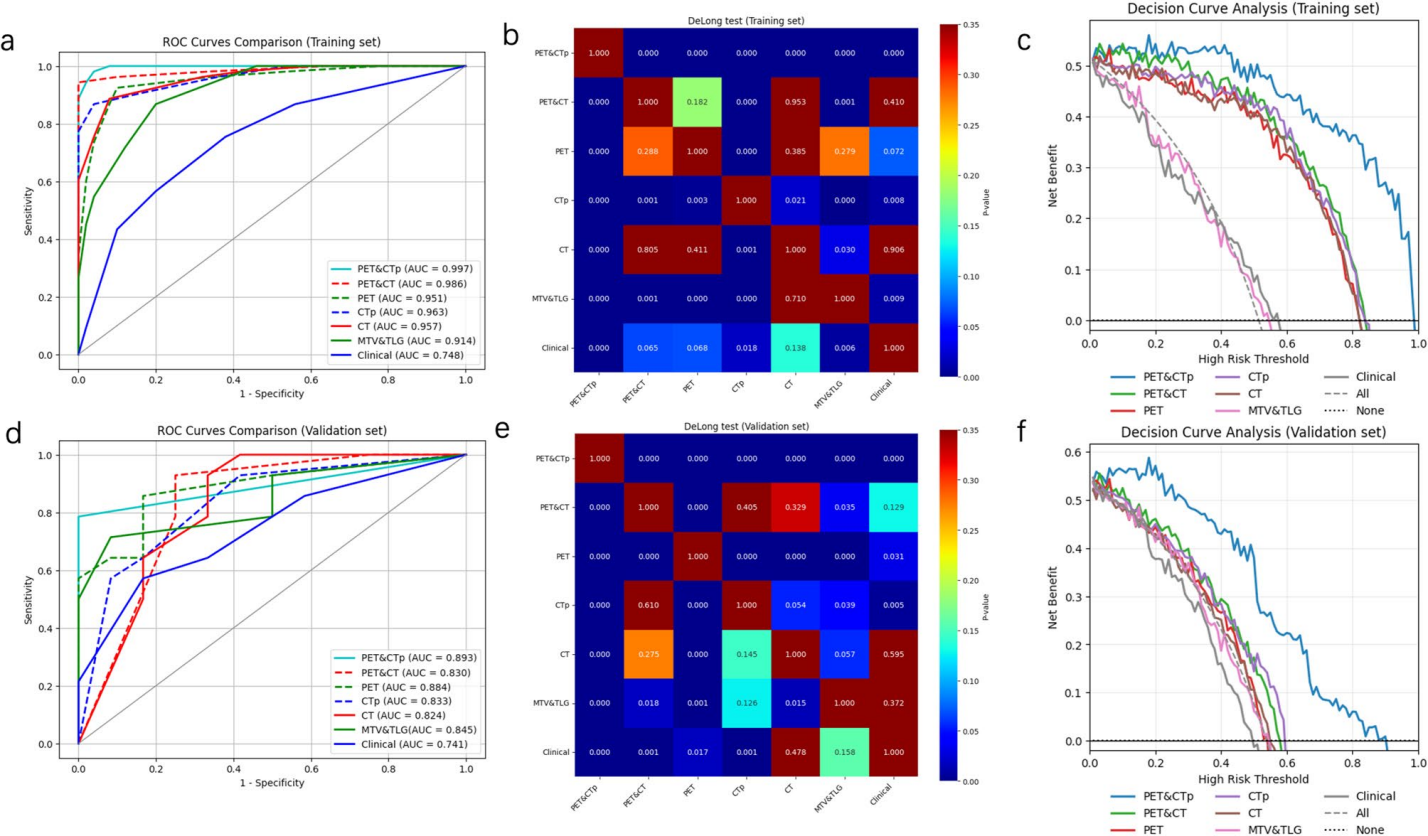
Model Performance Evaluation with DT-based models The receiver operating characteristic (ROC) curves, the DeLong test, and decision curve analysis (DCA) of all models in the training cohort (**a**,** b**,** c**), and validation cohort (**d**,** e**,** f**)

Fourty-two candidate models were constructed based on cross-combination of six feature selection methods (five radiomic models above-mentioned and clinical features) with six machine learning classifiers(SVM, DT, RF, Extra Trees, XGBoost, LR) to determine which model was optimal for HRCAs prediction and the results of train and validation can be seen in the supplement Tables 2, 3, 4, 5 and 6. Among these six categories of machine learning models, the DT based PET&CTp model, which had the highest AUC of 0.8929, was considered the optimal models and models performance were compared in different classifiers by DeLong test(See Supplement Table S7). The performance of DT-based five radiomic models were summarized in Table 3 and illustrated in Fig. 3. Notably, single-modality PET- features based on DT (AUC = 0.8839, 95% CI: 0.7290–0.9881) outperformed hybrid PET&CT approaches (AUC = 0.8304, 95% CI: 0.6569–0.9771). Among DT-based radiomic models, the PET&CTp fusion model yielded superior performance and all the radiomic models surpassing the clinical model benchmark (AUC = 0.7411, 95% CI: 0.5212–0.9033). DeLong test (*p* < 0.0001) and DCA confirmed the superior discriminative capacity and clinical utility of the DT-based PET&CTp model in the validation cohort (Fig. 3).

**Table 3:** Model diagnostic performance constructed by DT of six different feature set

Cohorts	Feature set	AUC (95%CI)	Acc	Sen	Spe	PPV	NPV	F1 score
Training set	PET&CTp	**0.9972(0.9911-1.0000)**	**0.9709**	0.9811	0.9600	0.963	0.9796	0.9720
	PET&CT	0.9857(0.9630-1.0000)	0.9709	0.9434	1.0000	1.0000	0.9434	0.9709
	PET	0.9513(0.9085–0.9851)	0.9126	0.9245	0.9000	0.9074	0.9184	0.9159
	CTp	0.9626(0.9282–0.9887)	0.9126	0.8679	0.9600	0.9583	0.8727	0.9109
	CT	0.9574(0.9189–0.9832)	0.9029	0.8868	0.9200	0.9216	0.8846	0.9039
	MTV&TLG	0.9140(0.8593–0.9581)	0.835	0.8679	0.8	0.8214	0.8511	0.844
	Clinical	0.7479(0.6545–0.8352)	0.6893	0.7547	0.6200	0.6780	0.7045	0.7143
Validation set	PET&CTp	**0.8929(0.7727-1.0000)**	**0.8846**	0.7857	1.0000	1.0000	0.8000	0.8800
	PET&CT	0.8304(0.6569–0.9771)	0.8462	0.9286	0.7500	0.8125	0.9000	0.8667
	PET	0.8839(0.7290–0.9881)	0.8462	0.8571	0.8333	0.8571	0.8333	0.8571
	CTp	0.8333(0.6666–0.9608)	0.7692	0.9286	0.5833	0.7222	0.8750	0.8125
	CT	0.8244(0.6343–0.9762)	0.8077	0.9286	0.6667	0.7647	0.8889	0.8387
	TLG&MTV	0.8452(0.6745–0.9818)	0.8077	0.7143	0.9167	0.9091	0.7333	0.8
	Clinical	0.7411(0.5212–0.9033)	0.6923	0.5714	0.8333	0.8000	0.6250	0.6666

ACC accuracy, AUC the area under the curve, 95%CI 95% confidence interval, PPV positive predictive value, NPV negative predictive value. The bold values mean the performance parameters of the best model among all the models in the training set and validation set, respectively

### Visual interpretation of the optimal model

**Fig. 4:**
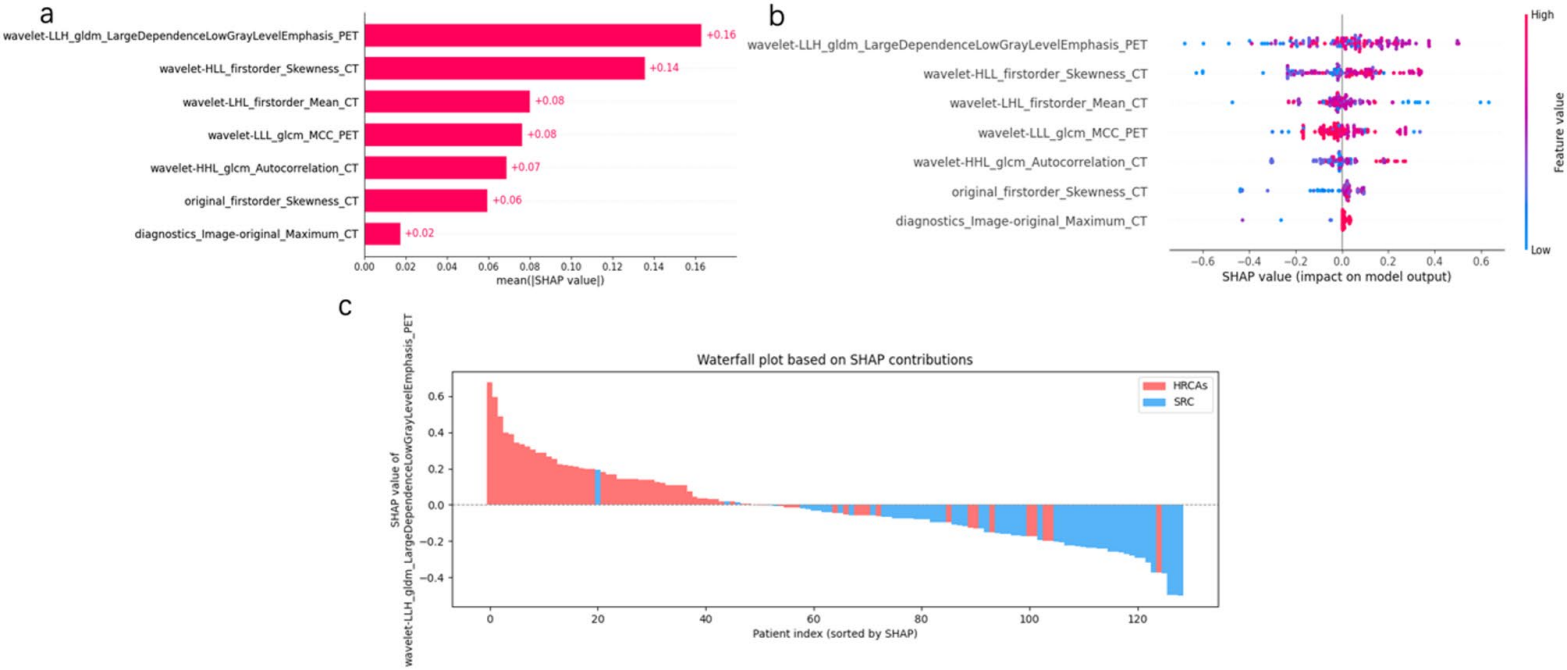
SHAP plot of DT-based PET&CTp model Fig. 4**a** A bar chart to show the contribution of each parameter to the model, and we can see that the most important factor in the image is the “wavelet-LLH_gldm_largeDependencelowGrayLevelEmphasis_PET”from PET. Figure 4**b** SHAP summary plot give a birds-eye view of feature importance on the model. The waterfall chart of Fig. 4**c** shows the distribution characteristics of the most importance indicator in the HRCAs group and the SRC group

To enhance model interpretability, SHAP were employed. The SHAP summary plot (Fig. 4a) ranked feature contributions within the DT-based PET&CTp model, with the most impactful being the PET-derived “wavelet-LLH_gldm_largeDependencelowGrayLevelEmphasis_PET” (mean |SHAP| = 0.16), followed by CT-based “wavelet-HLL_first-order_skewness”. SHAP dependence and waterfall plots (Fig. 4b and c) demonstrated that elevated values of “wavelet-LLH_gldm_largeDependencelowGrayLevelEmphasis_PET” were positively correlated with HRCAs classification probability, highlighting their importance in model decision-making.

### Survival stratification based on radiomics signature

**Fig. 5:**
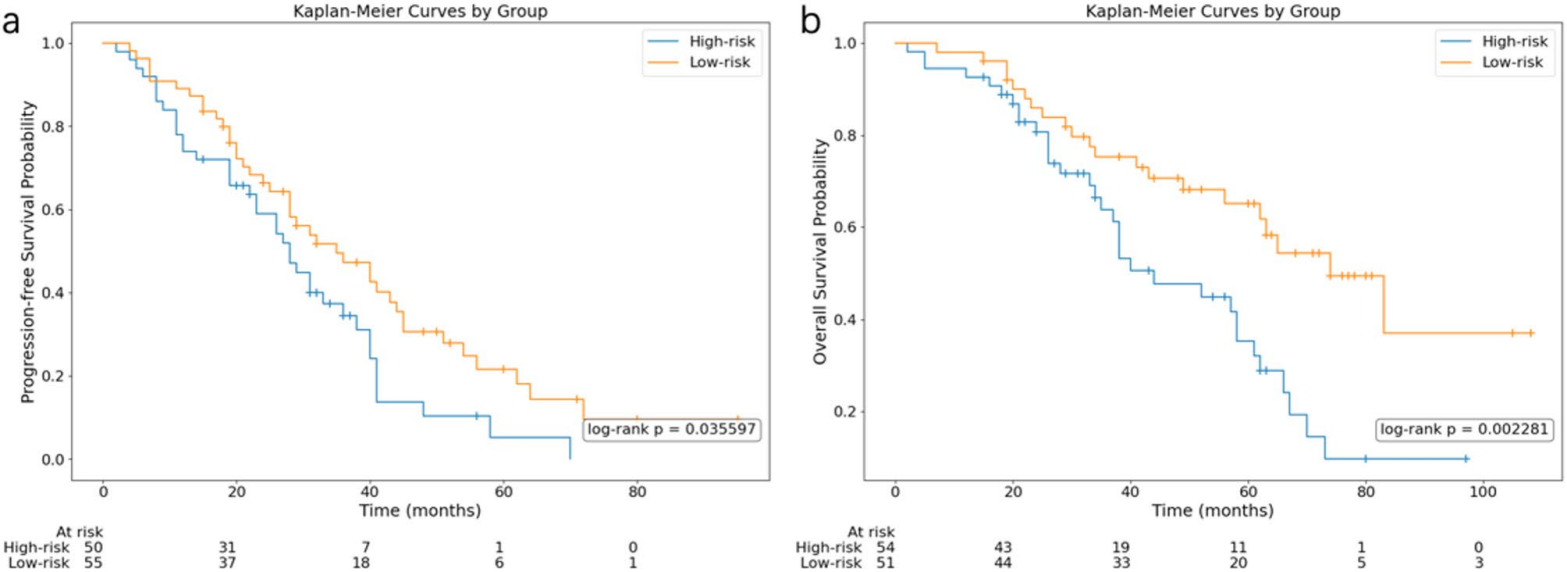
Survival Analysis Stratified by DT-based PET&CTp Model Score Kaplan-Meier analysis showed that, the high-risk and low-risk groups divided according to the output probability of the best model found significant difference in PFS and OS

Using the output probabilities from the optimal DT-based PET&CTp model to predict PFS, the study cohort (*n* = 105 with complete survival data) was stratified into high-risk (*n* = 50) and low-risk (*n* = 55) groups based on an optimised threshold of 0.7500. To predict OS, the study cohort (*n* = 105 with.

complete survival data) was stratified into high-risk (*n* = 54) and low-risk (*n* = 51) groups based on an optimised threshold of 0.6667. The high-risk group had a median PFS of 24.5 months and a median OS of 33.5 months, whereas the low-risk group demonstrated a median PFS of 29 months and OS of 50 months. Kaplan–Meier survival analysis revealed significant stratification for PFS (*p* = 0.035) and OS (*p* = 0.002) between the two groups indicating predictive capability of the model (Fig. 5a and b).

## Discussion

In this study, we developed multi-modal radiomic models for preoperative prediction of cytogenetic abnormality (CA) status in multiple myeloma (MM) using PET/CT imaging. The optimal model—the DT classifier integrating combined PET and CT radiomic features extracted from PET-visible lesions—demonstrated superior performance in discriminating HRCAs from SRC. Validation analyses confirmed its robustness (AUC: 0.89). Notably, models based on PET-visible lesions (PET&CTp) outperformed those using features from separately defined PET- and CT-visible lesions (PET&CT), suggesting that radiomic signatures from metabolically active regions more closely reflect cytogenetic profiles and underlying biological aggressiveness. The PET metric model (MTV&TLG) demonstrated predictive performance second only to the combined PET&CTp model and the PET radiomics model, but outperformed all other CT-based and clinical models, further validating the substantial predictive value of metabolically active PET lesions for determining cytogenetic status, with radiomic features exhibiting superior predictive value compared to conventional PET parameters. SHAP plots identified the most influential radiomic features, with their directional impacts quantified to enhance model transparency. Critically, risk stratification derived from this model significantly discriminated overall survival outcomes (log-rank *p* < 0.05), establishing CA status as a key prognostic determinant. This approach shows promise for clinical deployment in guiding risk-adapted MM management.

PET/CT is increasingly utilized in multiple myeloma (MM) and has been recommended by the IMWG as the preferred imaging modality for baseline staging and treatment response assessment, particularly in newly diagnosed patients [14, 16, 17, 38]. While several studies have evaluated the prognostic value of radiomics from 18F-FDG-PET/CT [17, 24, 26], non-invasive prediction of CA status—a critical determinant of therapeutic strategy and prognosis [12]—remains largely unexplored. Currently, CA profiling relies exclusively on invasive bone marrow biopsies, which are susceptible to sampling bias due to tumor heterogeneity [31] and fail to capture global disease burden. This approch limitation frequently leads to inadequate risk stratification in frail patients unable to tolerate repeated procedures, potentially compromising treatment selection. To address this unmet clinical need, we developed a novel multimodal model integrating baseline PET/CT radiomics to predict HRCA. To our knowledge, this represents the first study leveraging PET/CT radiomics for CA risk stratification in MM, with a substantially larger cohort (*n* = 129) than prior MRI-based attempts (*n* = 31–103) [27, 29, 30].

The radiomics model significantly outperformed the clinical model (AUC 0.89 vs. 0.74, *p* < 0.05). This improvement may be attributed to our dual-modality approach: systematic delineation of all PET/CT lesions enabled comprehensive capture of metabolic-anatomical heterogeneity, whereas prior MRI studies focused solely on spinal segments [27, 28, 30] using one or more MRI sequences. Through a metabolically-aligned feature fusion framework (combining PET and CT radiomics), we achieved superior performance (AUC = 0.89) compared to MRI-based models (AUC 0.84). Methodologically, we rigorously evaluated 42 candidate models, with the optimal DT-based models selected via DeLong testing.

Given the substantial spatial and functional discordance between PET and CT imaging manifestations, dual-modality lesion contouring was implemented to holistically quantify total tumor burden. This approach aligns with established evidence designating PET-positive lesions as metabolically active disease foci with proven prognostic significance [18, 39]. Critically, volumetric assessment of these active lesions correlates with adverse outcomes [18, 40], where bulky disease reflects baseline tumor burden prior to therapy. Radiomic features derived from PET masks exhibited superior predictive performance compared to those from CT. This observation is consistent with the biological relevance of FDG-PET, as elevated glucose uptake in metabolically active lesions may reflect alterations in the tumor microenvironment associated with cytogenetic aberrations [15]. SHAP value visualization further underscored the higher contribution of PET-derived features over CT-based ones. In particular, the strong predictive role of the PET wavelet feature—which reflects horizontal texture correlation in high-frequency domains—suggests its association with disrupted glucose metabolism in high-risk cytogenetic abnormalities (HRCAs). Nevertheless, the exact biological basis of radiomic features remains incompletely elucidated, and further histopathological correlation is warranted to validate these findings [41]. This validates the heightened biological relevance of metabolic radiomics in capturing cytogenetic profiles—likely mediated by their ability to decode tumor aggressiveness rooted in genetic aberrations [42].

Multivariable analysis identified serum β₂-microglobulin > 5500 µg/L, albumin < 35 g/L, and light-chain MM subtype as independent predictors of HRCAs (clinical model AUC = 0.74). These parameters—validated prognosticators in the ISS stage—are associated with adverse outcomes [9], with β₂-microglobulin > 5.5 mg/L recently reclassified as a high-risk criterion in updated MM stratification [43]. Light-chain MM portends worse survival than non-light-chain subtypes. Critically, our findings establish these factors as novel determinants of HRCAs. Although this clinical model showed modest predictive efficacy compared to radiomics (AUC: radiomics 0.89 vs. clinical 0.74), it retains complementary value. The model based on conventional PET/CT parameters demonstrated inferior performance compared to both the PET and PET&CTp radiomics models, yet remained superior to those relying solely on CT features. This suggests the value of metabolic lesion characteristics in representing disease status in patients. Conventional PET/CT metrics underperformed compared to radiomic models, which may be attributed to their reflection of global metabolic activity within the lesion rather than capturing the underlying spatial heterogeneity at a microscopic level [44]. In contrast, radiomics enables the quantification of subregional textural patterns and intralesional heterogeneity, potentially offering deeper insights into tumor biology [41].

In contrast, radiomics quantitatively extracts high-dimensional features that encode spatial heterogeneity and microenvironmental variations at the lesion level [31, 45], providing granular biomarkers directly linked to cytogenetic pathophysiology. The prognostic value of our integrated PET&CTp model, based on multiscale texture and morphology, adds biological plausibility to the radiomic features, underscoring their capacity to decode distinct phenotypic expressions of the disease [41]. Landmark analysis revealed significant PFS and OS divergence between risk strata defined by the model’s HRCA probability (median PFS: high-risk 24.5 months vs. low-risk 29 months; *p* = 0.0360; median OS: high-risk 33.5 months vs. low-risk 50 months; *p* = 0.0023). The observed PFS and OS stratification aligns with established evidence that HRCAs drive inferior survival [5, 12, 43]. Critically, this confirms that: CA status prediction models enable clinically relevant risk stratification; PET/CT radiomics captures prognostic biological aggressiveness beyond HRCAs. These findings position radiomics as a superior approach to conventional PET/CT parameters, providing multidimensional insights into tumor heterogeneity for precision treatment allocation.

Despite the promising results of this study, several limitations should be acknowledged. First, the sample size of 129 patients may be insufficient to draw definitive conclusions, particularly given the inherent heterogeneity of multiple myeloma and the complexity of radiomic models. Second, as a retrospective study conducted at a single institution, the findings may be influenced by selection bias and limited generalizability. Third, due to the constrained sample size, we were unable to perform subtype-specific analyses for individual HRCAs, which represents an important avenue for future research. Although internal validation indicated reasonable model stability, these limitations underscore the exploratory nature of our findings. Therefore, future prospective, multi-institutional studies with larger cohorts are essential to validate and generalize our model. Furthermore, while the radiomic signature demonstrated predictive value for HRCAs, the biological mechanisms underlying the association between imaging features and genetic profiles remain unclear. Future studies integrating radiomics with genomic and pathologic data are warranted to elucidate these relationships.

## Conclusion

In summary, this study established a PET/CT-based radiomic model for predicting cytogenetic abnormalities in multiple myeloma, demonstrating promising performance in identifying high-risk profiles and aiding risk stratification. However, the single-center nature and limited sample size constrain the generalizability of the findings. This work should be regarded as preliminary, highlighting the feasibility of the approach rather than providing definitive evidence. Further validation through larger, multi-center prospective studies is essential to confirm its clinical utility for personalized treatment in MM.

## Supplementary Information

Supplementary Material 1

## Data Availability

The datasets generated during and/or analyzed during the current study are available from the corresponding author on reasonable request.

## Abbreviations

MM: Multiple myeloma
HRCAs: High-risk cytogenetic abnormalities
^18^F-FDG PET/CT: ^18^F-fluoro-deoxyglucose positron emission tomography/computed tomographic
AUC: Area Under the Curve
OS: Overall survival
FISH: Fluorescence in situ hybridization
IMWG: International Myeloma Working Group
FLs: Focal lesions
EMD: Extramedullary disease
SUVmax: Maximum uptake values
MTV: Metabolic tumor volume
TLG: Total lesion glycolysis
PFS: Progression-free survival
NDMM: New diagnosis multiple myeloma
SUVmean: Mean standardized uptake value
SRC: Standard risk cytogenetic
ISS: International Staging System
EANM: European Association of Nuclear Medicine
LASSO: The least absolute shrinkage and selection operator
SVM: Support vector machine
DT: Decision tree
RF: Random forest
Extra Trees: Extremely randomized trees
LR: Logistic regression
ROC: Receiver operating characteristic
PPV: Positive predictive value
NPV: Negative predictive value
DCA: Decision curve analysis
SHAP: SHapley Additive exPlanations
LDH: Lactate dehydrogenase
LC: Light-chain
CA: Cytogenetic abnormality
