# Supplementary material for: A feasibility study of [18F] FDG PET/CT radiomics in predicting high-risk cytogenetic abnormalities in multiple myeloma
Source: EJNMMI Res. 2025 Oct 15;15:131. doi: 10.1186/s13550-025-01321-8 (PMC12528638; doi:10.1186/s13550-025-01321-8)
Supplement: Supplementary file 1 — Supplementary Material 1 [file 13550_2025_1321_MOESM1_ESM.docx]

**SUPPLEMENTAL MATERIALS**

**A Feasibility Study of [18F] FDG PET/CT Radiomics in Predicting High-Risk Cytogenetic Abnormalities in Multiple Myeloma**

Hong Chen^1^, Jinxin Han^2^, Haozhe Huang^3^, Qi He^4^, Xinqi Ren,^1^ Fan Yu^1^, Chunkang Chang^4^, Xuehai Ding^2^, Quanyong Luo^1^

*Hong Chen, Jingxin Han, Haozhe Huang and Qi He contributed equally to this work.*

*Corresponding authors: Chunkang Chang, Xuehai Ding, Quanyong Luo*

^1^Department of Nuclear Medicine, Shanghai Sixth People's Hospital Affiliated to Shanghai Jiao Tong University School of Medicine, Shanghai 200233, China.

^2^School of Computer Engineering and Science, Shanghai University, Shanghai 200444, China

^3^Department of Interventional Radiology, Fudan University Shanghai Cancer Center, Shanghai, China.

^4^Department of Hematology, Shanghai Sixth People's Hospital Affiliated to Shanghai Jiao Tong University School of Medicine, Shanghai 200233, China.

**Corresponding Authors**

Chunkang Chang, Department of Hematology, Shanghai Sixth People's Hospital Affiliated to Shanghai Jiao Tong University School of Medicine, 600 Yishan Road,Shanghai 200233, China

Xuehai Ding, School of Computer Engineering and Science, Shanghai University, Shanghai 200444, China

Quanyong Luo, Department of Nuclear Medicine, Shanghai Sixth People's Hospital Affiliated to Shanghai Jiao Tong University School of Medicine, 600 Yishan Road, Shanghai, 200233, China

**E-mail address of the corresponding author**: [luoqy@sjtu.edu.cn](mailto:luoqy@sjtu.edu.cn)

**SUPPLEMENTAL MATERIALS**

**PART I 1.1 PET/CT Co-registration：Image Resampling and Spatial Normalization**

The resampling process was implemented using the ResampleImageFilter from SimpleITK (version 2.4.0). The CT image was set as the reference image, and linear interpolation (sitkLinear) was selected as the interpolation method to balance the smoothness of continuous values and computational efficiency.

During resampling, the output PET images were configured to have identical geometric attributes as the reference CT image, including voxel spacing, size, origin, and direction cosine matrix, thereby ensuring complete spatial registration between the two modalities. An identity transform was applied throughout the process, meaning no additional non-rigid deformation was introduced—only geometric parameter-based resampling was performed.

As a result, all PET images were processed to exhibit the same spatial resolution and dimensions as their corresponding CT images, facilitating subsequent multimodal image analysis and fusion.

1.2 Metabolic Tumor Volume (MTV) and Total Lesion Glycolysis (TLG) Calculation Methods

Metabolic Tumor Volume (MTV) was calculated by first identifying connected components within the segmentation mask for each lesion. The number of voxels per lesion, denoted as N_l_, was multiplied by the voxel volume v_voxel, which was derived from the diagonal elements of the PET image affine matrix to obtain the voxel resolution (Δx,Δy,Δz). The voxel volume was computed as v_voxel=Δx⋅Δy⋅Δz/1000 (cm³). Thus, the MTV for a lesion lwas defined as:

MTV(l)=N_l_×v_voxel.

This metric objectively reflects the three-dimensional metabolic burden of the lesion.

Total Lesion Glycolysis (TLG) was obtained by first extracting the SUV values within the mask(n) of each lesion and computing the mean SUV value, SUVmean(l). The TLG was then calculated as the product of the mean SUV and the corresponding MTV(l):

TLG=SUVmean(1) ×MTV(1)+ SUVmean(2)×MTV(2)+……SUVmean (l) ×MTV(l).

TLG integrates both metabolic intensity and volumetric extent into a single measure, providing a comprehensive quantification of the total glycolytic activity of the lesion.

**PART II Radiomics Analysis**

2.1 Feature Extraction from PET and/or CT mask:

Radiomic features were extracted from medical images (nii.gz format) using PyRadiomics. This yielded a total of 851 features from the PET/CT mask. Feature Extraction Strategy as follow: To address spatial misalignment between CT and PET in multiple myeloma, five feature categories were derived:

(i) CT model: CT radiomic features derived from CT visible lesions region

(ii) CTp model: CT radiomic features derived from co-registered PET visible lesions region

(iii) PET model: PET radiomic features derived from PET visible lesions region

(iv) PET&CT model: constructed by utilizing the radiomic features derived from PET masks and CT visible lesions region of both imaging modalities, separately

(v) PET&CTp model: established by integrating radiomic features from PET visible lesions region of both imaging modalities

2.2 Feature Standardization:

The extracted radiomic features underwent Z-score normalization to transform the data to follow a standard normal distribution (N~ (0, 1)).

2.3. Feature Selection via Correlation Analysis:

Pairwise Pearson correlation coefficients were computed between all features. To mitigate redundancy, features exhibiting a correlation coefficient exceeding 0.9 were identified. From each highly correlated pair (r > 0.9), only one feature was retained, resulting in a reduced set of features.

2.4. Feature Dimensionality Reduction using Least Absolute Shrinkage and Selection Operator (LASSO):

LASSO regression with five-fold cross-validation was applied for further feature selection. The optimal penalty coefficient (λ) was determined. This process selected final predictive features.

All image features extraction and screening results seeTable S1.

Table S1 All image features extraction and screening results

| Mask | Number of total Radiomic features | Number of total Radiomic features After Correlation Analysis | optimal penalty coefficient (λ) | final predictive features |
| --- | --- | --- | --- | --- |
| CT | 851 | 178 | 9.1201 | 8 |
| CTp | 851 | 150 | 8.7096 | 8 |
| PET | 851 | 189 | 7.7625 | 9 |
| PET&CT | 851*2 | 367 | 10.839 | 8 |
| PET&CTp | 851*2 | 310 | 9.9770 | 7 |

**2.5 Randomized data partitioning with performance-based selection:**
Repeated randomized data splitting was performed, with the optimal partition (yielding the highest model performance) retained for subsequent analysis.

**2.6 Multi-model machine learning framework:**
Multiple machine learning algorithms were employed for model training, including: Logistic Regression (LR), Support Vector Machines (SVM), Decision Trees (DT), Random Forests (RF), Extremely Randomized Trees (ExtraTrees) ,eXtreme Gradient Boosting (XGBoost).Diagnostic performance of five different radiomic models (CT, CTp, PET, PET&CT, PET&CTp) with six classifiers in the training and validation cohorts are presented in Table S2,. Table S3, Table S4, Table S5, Table S6 respectively.

**2.7 Model validation encompassed**: discriminative performance (AUC, F1-score, accuracy, sensitivity, specificity, positive predictive value (PPV), and negative predictive value (NPV))（Table S2-Table S6）, comparative analysis (DeLong test) presented in Table S7.

Table S2 CT radiomic features diagnostic performance of six different classifiers in the training and validation cohorts

| Cohorts | Models | AUC (95 %CI) | Acc | Sen | Spe | PPV | NPV | F1 score |
| --- | --- | --- | --- | --- | --- | --- | --- | --- |
| Training Set | LR | 0.6453(0.5436-0.7544) | 0.6602 | 0.4528 | 0.88 | 0.8 | 0.6027 | 0.5783 |
|  | SVM | 0.6087(0.5000-0.7173) | 0.6117 | 0.3208 | 0.92 | 0.8095 | 0.561 | 0.4595 |
|  | DT | 0.9574(0.9189-0.9832) | 0.9029 | 0.8868 | 0.92 | 0.9216 | 0.8846 | 0.9039 |
|  | RF | 0.9566(0.9145-0.9876) | 0.8932 | 0.9623 | 0.82 | 0.85 | 0.9535 | 0.9027 |
|  | ExtraTree | 0.9298(0.8770-0.9717) | 0.8447 | 0.9434 | 0.74 | 0.7937 | 0.925 | 0.8621 |
|  | XGBoost | 0.9545(0.9191-0.9849) | 0.9029 | 0.9245 | 0.88 | 0.8909 | 0.9167 | 0.9074 |
| Validation Set | LR | 0.6190(0.3868-0.8393) | 0.6538 | 0.3571 | 1 | 1 | 0.5714 | 0.5263 |
|  | SVM | 0.5923(0.3549-0.8212) | 0.6538 | 0.4286 | 0.9167 | 0.8571 | 0.5789 | 0.5714 |
|  | DT | **0.8244(0.6343-0.9762)** | 0.8077 | 0.9286 | 0.6667 | 0.7647 | 0.8889 | 0.8387 |
|  | RF | 0.5417(0.3029-0.7811) | 0.5769 | 0.3571 | 0.8333 | 0.7143 | 0.5263 | 0.4762 |
|  | ExtraTree | 0.6310(0.3869-0.8424) | 0.6923 | 0.7857 | 0.5833 | 0.6875 | 0.7 | 0.7333 |
|  | XGBoost | 0.5476(0.3124-0.7821) | 0.6154 | 0.8571 | 0.3333 | 0.6 | 0.6667 | 0.7059 |

Table S3 CTp radiomics features diagnostic performance of six different selected classifiers in the training and validation cohorts

| Cohorts | Models | AUC (95 %CI) | Acc | Sen | Spe | PPV | NPV | F1 score |
| --- | --- | --- | --- | --- | --- | --- | --- | --- |
| Traning Set | LR | 0.6540(0.5491-0.7586) | 0.6602 | 0.4528 | 0.88 | 0.8 | 0.6027 | 0.5783 |
|  | SVM | 0.4558(0.3450-0.5604) | 0.5437 | 0.2453 | 0.86 | 0.65 | 0.5181 | 0.3562 |
|  | DT | 0.9626(0.9282-0.9887) | 0.9126 | 0.8679 | 0.96 | 0.9583 | 0.8727 | 0.9109 |
|  | RF | 0.9400(0.8927-0.9781) | 0.8835 | 0.9623 | 0.8 | 0.8361 | 0.9524 | 0.8948 |
|  | ExtraTree | 0.9370(0.8882-0.9736) | 0.8544 | 0.8491 | 0.86 | 0.8654 | 0.8431 | 0.8572 |
|  | XGBoost | 0.9623(0.9270-0.9877) | 0.8932 | 0.9245 | 0.86 | 0.875 | 0.9149 | 0.8991 |
| Validation Set | LR | 0.6548(0.4242-0.8486) | 0.6923 | 0.9286 | 0.4167 | 0.65 | 0.8333 | 0.7647 |
|  | SVM | 0.4405(0.2130-0.6667) | 0.5385 | 0.1429 | 1 | 1 | 0.5 | 0.2501 |
|  | DT | **0.8333(0.6666-0.9608)** | 0.7692 | 0.9286 | 0.5833 | 0.7222 | 0.875 | 0.8125 |
|  | RF | 0.6786(0.4665-0.8869) | 0.6923 | 0.6429 | 0.75 | 0.75 | 0.6429 | 0.6923 |
|  | ExtraTree | 0.7619(0.5562-0.9333) | 0.7308 | 0.5714 | 0.9167 | 0.8889 | 0.6471 | 0.6956 |
|  | XGBoost | 0.5714(0.3312-0.7938) | 0.5769 | 0.2143 | 1 | 1 | 0.5217 | 0.353 |

Table S4 PET Radiomics features diagnostic performance of six different selected classifiers in the training and validation cohorts

| Cohorts | Models | AUC (95 %CI) | Acc | Sen | Spe | PPV | NPV | F1 score |
| --- | --- | --- | --- | --- | --- | --- | --- | --- |
| Traning Set | LR | 0.7317(0.6264-0.8223) | 0.699 | 0.434 | 0.98 | 0.9583 | 0.6203 | 0.5974 |
|  | SVM | 0.6315(0.5173-0.7374) | 0.6214 | 0.3774 | 0.88 | 0.7692 | 0.5714 | 0.5064 |
|  | DT | 0.9513(0.9085-0.9851) | 0.9126 | 0.9245 | 0.9 | 0.9074 | 0.9184 | 0.9159 |
|  | RF | 0.9808(0.9570-0.9973) | 0.9417 | 0.9245 | 0.96 | 0.9608 | 0.9231 | 0.9423 |
|  | ExtraTree | 0.9860(0.9687-0.9972) | 0.9515 | 1 | 0.9 | 0.9138 | 1 | 0.955 |
|  | XGBoost | 0.9815(0.9567-0.9970) | 0.932 | 0.8868 | 0.98 | 0.9792 | 0.8909 | 0.9307 |
| Validation Set | LR | 0.6012(0.3562-0.8182) | 0.6538 | 0.4286 | 0.9167 | 0.8571 | 0.5789 | 0.5714 |
|  | SVM | 0.4583(0.2450-0.6994) | 0.5769 | 0.3571 | 0.8333 | 0.7143 | 0.5263 | 0.4762 |
|  | DT | **0.8839(0.7290-0.9881)** | 0.8462 | 0.8571 | 0.8333 | 0.8571 | 0.8333 | 0.8571 |
|  | RF | 0.7083(0.4821-0.8869) | 0.6923 | 0.5 | 0.9167 | 0.875 | 0.6111 | 0.6364 |
|  | ExtraTree | 0.7560(0.5413-0.9248) | 0.7308 | 0.5714 | 0.9167 | 0.8889 | 0.6471 | 0.6956 |
|  | XGBoost | 0.6250(0.3875-0.8452) | 0.6538 | 0.7143 | 0.5833 | 0.6667 | 0.6364 | 0.6897 |

Table S5 PET&CT radiomics features diagnostic performance of six different selected classifiers in the training and validation cohorts

| Cohorts | Models | AUC (95 %CI) | | Acc | Sen | Spe | PPV | NPV | F1 score |
| --- | --- | --- | --- | --- | --- | --- | --- | --- | --- |
| Traning Set | LR | | 0.6879(0.5831-0.7938) | 0.6796 | 0.6226 | 0.74 | 0.7174 | 0.6491 | 0.6666 |
|  | SVM | | 0.4383(0.3393-0.5493) | 0.5146 | 0.1698 | 0.88 | 0.6 | 0.5 | 0.2647 |
|  | DT | | 0.9857(0.9630-1.0000) | 0.9709 | 0.9434 | 1 | 1 | 0.9434 | 0.9709 |
|  | RF | | 0.9253(0.8751-0.9645) | 0.8544 | 0.7736 | 0.94 | 0.9318 | 0.7966 | 0.8454 |
|  | ExtraTree | | 0.9536(0.9166-0.9811) | 0.8835 | 0.8302 | 0.94 | 0.9362 | 0.8393 | 0.88 |
|  | XGBoost | | 0.9442(0.9075-0.9785) | 0.8641 | 0.7925 | 0.94 | 0.9333 | 0.8103 | 0.8572 |
| Validation Set | LR | | 0.7143(0.5000-0.9091) | 0.7308 | 0.7143 | 0.75 | 0.7692 | 0.6923 | 0.7407 |
|  | SVM | | 0.3214(0.1250-0.5656) | 0.5 | 0.4286 | 0.5833 | 0.5455 | 0.4667 | 0.48 |
|  | DT | | **0.8304(0.6569-0.9771)** | 0.8462 | 0.9286 | 0.75 | 0.8125 | 0.9 | 0.8667 |
|  | RF | | 0.6786(0.4484-0.8848) | 0.6923 | 0.7857 | 0.5833 | 0.6875 | 0.7 | 0.7333 |
|  | ExtraTree | | 0.7857(0.5621-0.9543) | 0.7692 | 0.8571 | 0.6667 | 0.75 | 0.8 | 0.8 |
|  | XGBoost | | 0.6667(0.4319-0.8810) | 0.7308 | 0.9286 | 0.5 | 0.6842 | 0.8571 | 0.7879 |

Table S6 PET&CTp radiomic features Diagnostic performance of six different selected classifiers in the training and validation cohorts

| Cohorts | Models | AUC (95 %CI) | Acc | Sen | Spe | PPV | NPV | F1 score |
| --- | --- | --- | --- | --- | --- | --- | --- | --- |
| Traning Set | LR | 0.6879(0.5828-0.7834) | 0.6505 | 0.4906 | 0.82 | 0.7429 | 0.6029 | 0.5909 |
|  | SVM | 0.4174(0.3094-0.5307) | 0.4951 | 0.0189 | 1 | 1 | 0.4902 | 0.0371 |
|  | DT | 0.9972(0.9911-1.0000) | 0.9709 | 0.9811 | 0.96 | 0.963 | 0.9796 | 0.972 |
|  | RF | 0.9381(0.8919-0.9766) | 0.8932 | 0.9434 | 0.84 | 0.8621 | 0.9333 | 0.9009 |
|  | ExtraTree | 0.9079(0.8499-0.9578) | 0.8447 | 0.9245 | 0.76 | 0.8033 | 0.9048 | 0.8596 |
|  | XGBoost | 0.9289(0.8753-0.9738) | 0.8641 | 0.9434 | 0.78 | 0.8197 | 0.9286 | 0.8772 |
| Validation Set | LR | 0.6131(0.3725-0.8126) | 0.6154 | 0.2857 | 1 | 1 | 0.5455 | 0.4444 |
|  | SVM | 0.3750(0.1699-0.6062) | 0.5769 | 0.2857 | 0.9167 | 0.8 | 0.5238 | 0.421 |
|  | DT | **0.8929(0.7727-1.0000)** | 0.8846 | 0.7857 | 1 | 1 | 0.8 | 0.88 |
|  | RF | 0.6548(0.4241-0.8521) | 0.6538 | 0.5 | 0.8333 | 0.7778 | 0.5882 | 0.6087 |
|  | ExtraTree | 0.7083(0.4937-0.8909) | 0.6923 | 0.9286 | 0.4167 | 0.65 | 0.8333 | 0.7647 |
|  | XGBoost | 0.6161(0.3879-0.8274) | 0.6538 | 0.7143 | 0.5833 | 0.6667 | 0.6364 | 0.6897 |

Table S7 DeLong test（DT vs other 5 classifiers）

| DT vs model | CT | | CTp | | PET | | PET&CT | | PET&CTp | | |
| --- | --- | --- | --- | --- | --- | --- | --- | --- | --- | --- | --- |
|  | t | p-value | t | p-value | t | p-value | t | p-value | t | p-value |  |
| SVM | 6.04 | <0.001^*^ | 11.27 | <0.001^*^ | 11.33 | <0.001^*^ | 18.21 | <0.001^*^ | 28.71 | <0.001^*^ |  |
| LR | 18.30 | <0.001^*^ | 7.16 | <0.001^*^ | 16.81 | <0.001^*^ | 14.53 | <0.001^*^ | 12.76 | <0.001^*^ |  |
| RF | 2.35 | 0.0365^*^ | 10.87 | <0.001^*^ | 8.23 | <0.001^*^ | 2.19 | 0.0484^*^ | 6.65 | <0.001^*^ |  |
| ET | 2.37 | 0.0352^*^ | 6.13 | <0.001^*^ | 17.43 | <0.001^*^ | 3.59 | 0.0037^*^ | 7.38 | <0.001^*^ |  |
| XGB | 2.49 | 0.0281^*^ | 11.13 | <0.001^*^ | 5.71 | <0.001^*^ | 5.26 | <0.001^*^ | 7.07 | <0.001^*^ |  |

*t ,t-statistic, ^*^ statistically significant difference，LR,Logistic Regression, SVM, Support Vector Machines,DT,Decision Trees, RF,Random Forests, ET,Extremely Randomized Trees, XGB,eXtreme Gradient Boosting*

APRT III **Clinical Predictive models and PET metrics model**

Table S8 Clinical Features Diagnostic performance of six different selected models in the training and validation cohorts.

| Cohorts | Models | AUC (95 %CI) | Acc | Sen | Spe | PPV | NPV | F1 score |
| --- | --- | --- | --- | --- | --- | --- | --- | --- |
| Traning Set | LR | 0.7355(0.6427-0.8264) | 0.699 | 0.6038 | 0.8 | 0.7619 | 0.6557 | 0.6737 |
|  | SVM | 0.6411(0.5295-0.7447) | 0.6893 | 0.7358 | 0.64 | 0.6842 | 0.6957 | 0.7091 |
|  | DT | 0.7479(0.6545-0.8352) | 0.6893 | 0.7547 | 0.62 | 0.678 | 0.7045 | 0.7143 |
|  | RF | 0.7460(0.6494-0.8347) | 0.6893 | 0.7358 | 0.64 | 0.6842 | 0.6957 | 0.7091 |
|  | ExtraTree | 0.7309(0.6361-0.8204) | 0.6699 | 0.6415 | 0.7 | 0.6939 | 0.6481 | 0.6667 |
|  | XGBoost | 0.7475(0.6563-0.8362) | 0.6893 | 0.7547 | 0.62 | 0.678 | 0.7045 | 0.7143 |
| Validation Set | LR | 0.7113(0.4757-0.8917) | 0.6923 | 0.5 | 0.9167 | 0.875 | 0.6111 | 0.6364 |
|  | SVM | 0.5506(0.3253-0.7875) | 0.6538 | 0.8571 | 0.4167 | 0.6316 | 0.7143 | 0.7273 |
|  | DT | **0.7411(0.5212-0.9033)** | 0.6923 | 0.5714 | 0.8333 | 0.8 | 0.625 | 0.6666 |
|  | RF | 0.7054(0.4812-0.8948) | 0.6923 | 0.5714 | 0.8333 | 0.8 | 0.625 | 0.6666 |
|  | ExtraTree | 0.7232(0.5037-0.8938) | 0.6923 | 0.5714 | 0.8333 | 0.8 | 0.625 | 0.6666 |
|  | XGBoost | 0.7054(0.4812-0.8948) | 0.6923 | 0.5714 | 0.8333 | 0.8 | 0.625 | 0.6666 |

**Table S9** PET metrics model (MTV&TLG) Diagnostic performance of six different selected models in the training and validation cohorts.

| Cohorts | Models | AUC (95 %CI) | Acc | Sen | Spe | PPV | NPV | F1 score |
| --- | --- | --- | --- | --- | --- | --- | --- | --- |
| Traning Set | LR | 0.5434(0.4298-0.6554) | 0.5728 | 0.8302 | 0.3 | 0.557 | 0.625 | 0.6667 |
|  | SVM | 0.4543(0.3454-0.5644) | 0.534 | 0.717 | 0.34 | 0.5352 | 0.5312 | 0.6129 |
|  | DT | 0.9140(0.8593-0.9581) | 0.835 | 0.8679 | 0.8 | 0.8214 | 0.8511 | 0.844 |
|  | RF | 0.7906(0.6946-0.8730) | 0.7573 | 0.6226 | 0.9 | 0.8684 | 0.6923 | 0.7252 |
|  | ExtraTree | 0.8740(0.8095-0.9353) | 0.7961 | 0.7925 | 0.8 | 0.8077 | 0.7843 | 0.8 |
|  | XGBoost | 0.8798(0.8041-0.9384) | 0.7961 | 0.8302 | 0.76 | 0.7857 | 0.8085 | 0.8073 |
| Validation Set | LR | 0.4226(0.1938-0.6909) | 0.6154 | 0.3571 | 0.9167 | 0.8333 | 0.55 | 0.5 |
|  | SVM | 0.2976(0.0947-0.5444) | 0.5 | 0.0714 | 1 | 1 | 0.48 | 0.1333 |
|  | DT | **0.8452(0.6745-0.9818)** | 0.8077 | 0.7143 | 0.9167 | 0.9091 | 0.7333 | 0.8 |
|  | RF | 0.8333(0.6488-0.9909) | 0.8077 | 0.6429 | 1 | 1 | 0.7059 | 0.7826 |
|  | ExtraTree | 0.6488(0.4303-0.8750) | 0.6923 | 0.5714 | 0.8333 | 0.8 | 0.625 | 0.6666 |
|  | XGBoost | 0.8006(0.6061-0.9510) | 0.7692 | 0.5714 | 1 | 1 | 0.6667 | 0.7272 |
